# The era of cultivating smart rice with high light efficiency and heat tolerance has come of age

**DOI:** 10.3389/fpls.2022.1021203

**Published:** 2022-10-07

**Authors:** Qiuping Shen, Yujun Xie, Xinzhe Qiu, Jinsheng Yu

**Affiliations:** ^1^ The Key Laboratory for Quality Improvement of Agricultural Products of Zhejiang Province, Zhejiang A & F University, Hangzhou, China; ^2^ College of Advanced Agricultural Sciences, Zhejiang A & F University, Hangzhou, China

**Keywords:** smart rice, high light efficiency, C4 engineering, heat tolerance, breeding

## Abstract

How to improve the yield of crops has always been the focus of breeding research. Due to the population growth and global climate change, the demand for food has increased sharply, which has brought great challenges to agricultural production. In order to make up for the limitation of global cultivated land area, it is necessary to further improve the output of crops. Photosynthesis is the main source of plant assimilate accumulation, which has a profound impact on the formation of its yield. This review focuses on the cultivation of high light efficiency plants, introduces the main technical means and research progress in improving the photosynthetic efficiency of plants, and discusses the main problems and difficulties faced by the cultivation of high light efficiency plants. At the same time, in view of the frequent occurrence of high-temperature disasters caused by global warming, which seriously threatened plant normal production, we reviewed the response mechanism of plants to heat stress, introduced the methods and strategies of how to cultivate heat tolerant crops, especially rice, and briefly reviewed the progress of heat tolerant research at present. Given big progress in these area, the era of cultivating smart rice with high light efficiency and heat tolerance has come of age.

## Introduction

Due to the limited arable land worldwide, human beings have been committed to continuously improving the output of crops. Most of the yield accumulation of plants comes from the assimilation products of photosynthesis. The photosynthetic yield of any crop depends on the effective solar radiation, radiation capture efficiency and photosynthetic efficiency throughout the growing season (Jansson et al., 2018). Because of the continuous growth of the world’s population, it is expected that the global population will reach 9 billion by 2050, which means that the demand for food will rise sharply, bringing great challenges to agricultural production (Stratonovitch and Semenov, 2015). In order to meet the food requirement, the existing food production must increase by nearly 70% (Lobell et al., 2011) and increasing the yield accumulation of plants by various means will be the most important way.

High light efficiency refers to higher light utilization efficiency under low light and low CO_2_ concentration, or stronger photosynthesis than other plants under the same conditions. Although the current global CO_2_ concentration has increased, which seems beneficial to crop photosynthesis, this change is concentrated in the past few decades since 1975, and plants lack sufficient time to adapt to such rapid environmental changes. In addition, the decline of nitrogen uptake by plants under high CO_2_ concentration and the source-sink limitation also imply that the method of changing CO_2_ concentration to increase yield is not sustainable (Drake et al., 1997; Long et al., 2015; Zhu et al., 2021). Theoretically, the potential photosynthetic efficiencies of C3 and C4 plants were maximally able to reach 0.046 and 0.060, respectively. However, the current photosynthetic efficiencies of the two types of plants during the growing season only reach 47% and 57% of their theoretical values, respectively, and still have a large rising space (Zhu et al., 2010; Hein et al., 2021). In addition, the frequent occurrence of extreme high temperature caused by climate change in recent years has affected the normal production of a variety of crops, causing plant photosynthesis to be blocked and production to be reduced. It is estimated that the yield of rice will decrease by 3%-8% for every 1°C increase in the average temperature (Paul et al., 2020). This shows that, in addition to improve the photosynthetic efficiency of plants, it is also of great significance to ensure that plants can maintain a normal yield balance under the coming extreme high temperature. With the advent of post functional genomics in rice, breeding strategies that utilize beneficial alleles and eliminate deleterious alleles to optimize crop genome have broad prospects for designing future crops (Varshney et al., 2021; Yu et al., 2022), and the era of breeding smart rice with high light efficiency and heat resistance has come of age.

## Research progress of high light efficiency

Photosynthetic products are synthesized in leaves, transported to stems and leaf sheaths and stored as NSCs (non-structural carbohydrates), and then transferred to grains during grain filling (Hein et al., 2021). There are many factors affecting plant photosynthesis. Besides the main environmental conditions such as temperature and light, the morphological development and genetic regulation of plants are also important factors limiting their own photosynthesis. The improvement of photosynthetic rate of crop leaves can be achieved by targeted control of single component processes, such as improving the affinity of key enzyme Rubisco (1,5-ribose diphosphate carboxylation oxygenase) for CO_2_ (Long, 1991), increasing leaf vein density (Feldman et al., 2017), and adjusting stomatal size and density (Xiong et al., 2022). Furthermore, it is also an effective way to improve photosynthetic efficiency to explore key genes affecting photosynthesis through molecular genetic means.

According to the different carbon fixation products, plants can be divided into C3 and C4 types. Some important food crops such as rice and wheat belong to C3 plants, and their photosynthetic efficiencies largely limited by Rubisco’s oxygenation activity and photorespiration (Atkinson et al., 2016; Singer et al., 2019). In contrast, C4 plants can improve their photosynthetic efficiency by about 30% compared with C3 plants due to their extremely low photorespiration level and CO_2_ concentration mechanism (CCM, Langdale, 2011). Studies have shown that only 10% increase in photosynthetic efficiency can increase crop yield by nearly 50% (Zhu et al., 2008). It can be seen that if C4 pathway can be introduced into C3 plants such as rice, it will make great contributions to improve grain yield.

In addition, with the development of high-throughput phenotyping technology, people can accurately evaluate the phenotype of a large number of plants within a short time, which provides great convenience for breeding (Araus et al., 2018). In general, the leaves of plants can display different reflectance values at specific wavelengths due to different growth conditions. Therefore, the reflection wavelength of different plant leaves can be obtained by using reflection spectrum technology, which is very effective for determining various leaf characteristics including leaf greenness, light utilization efficiency and leaf pigment content (Tayade et al., 2022). At the same time, in view of the subtle color change of leaves caused by chlorophyll decomposition, reflection spectrum technology is also widely used in determining the senescence rate of leaves (Hein et al., 2021). This not only provides a rapid and accurate method for evaluating the photosynthetic efficiency of different plants, but also provides technical support for cultivating and screening plants with high photosynthetic efficiency, so it has great application potential.

## Design C4 crops

At present, people have made preliminary exploration in the design of C4 crops by means of genetic engineering (Ishimaru et al., 1998; Wang et al., 2012; Lian et al., 2014; Cheng et al., 2016; Kandoi et al., 2022). Many studies have shown that overexpression of key photosynthetic enzymes of C4 pathway helped to improve photosynthetic efficiency of C3 plants. However, this enhancement effect is not caused by the introduction of CCM, but by the increased chlorophyll synthesis caused by enhanced metabolism (Kandoi et al., 2022). Thus, the gained effect was quite limited. Given the effect of overexpression of individual photosynthetic enzymes on the rate of carbon assimilation, there are several attempts to combinatorically over-express multiple C4 photosynthetic enzymes to further enhance photosynthesis. However, the results showed that this approach not only did not concentrate CO_2_ near the chloroplast, but also produced a slight developmental delay (Häusler et al., 2001; Taniguchi et al., 2008; Yadav and Mishra, 2020). Since the current design for C4 crops still stays on single-cell methods such as overexpression of some photosynthetic genes, this limited improvement in photosynthetic efficiency can’t bring substantial improvement in yield (Cui, 2021).

The complex mechanism of C4 pathway is not only reflected in the types of photosynthetic enzymes. The spatial separation of C4 plant photosynthesis is the main reason for its formation of CCM (Furbank, 2017). Mesophyll cells tightly surround Bundle sheath (BS) cells in the center, making BS cells isolated from external O_2_ and reducing the occurrence of photorespiration. Moreover, BS cells are significantly larger than C3 plants and contain more chloroplasts, which greatly improves the photosynthetic efficiency of C4 plants (Yadav and Mishra, 2020).

**Figure 1:**
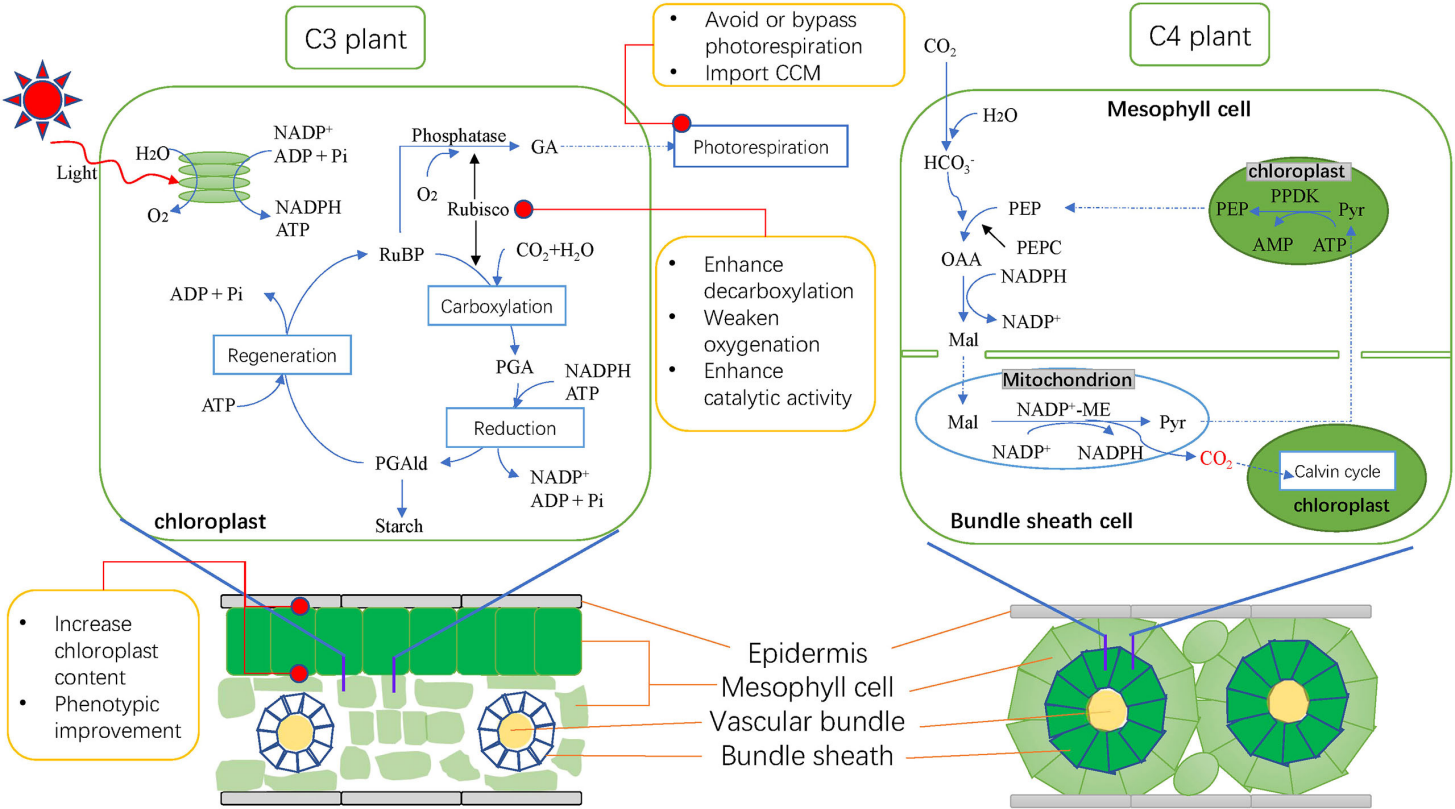
Photosynthetic pathway, leaf structure of C3 and C4 plants and main improvement methods for C3 plants at present. C3 plants on the left and C4 plants on the right. Blue solid line represents the photosynthetic reaction; blue dotted line represents the transport of substances; black line represents the catalysis of the enzyme; red dots represent major improvement objectives. RuBP, ribulose-1,5-bisphosphate; Rubisco, RuBP carboxylase/oxygenase; PGA, 3-phosphoglycerate; PGAld, 3-phosphoglyceraldehyde; GA, glycollic acid; PEP, Phosphoenolpyruvate; PEPC, Phosphoenolpyruvate carboxylase; OAA, oxaloacetic acid; Mal, malic acid; Pyr, pyruvic acid; NADP-ME, NADP-malic enzyme; PPDK, pyruvate phosphate dikinase; CCM, carbon concentration mechanism.

On the other hand, the unique Kranz structure of C4 plants’ leaves is another important feature that distinguishes C4 plants from C3 plants (**Figure 1**, Lundgren et al., 2014). The leaves of C4 plants have developed and more BS cells and more dense leaf vein tissues, and the connection between BS cells and mesophyll cells is also closer, which promotes the exchange of materials between them. Compared with C3 plants, C4 plants have higher material transport efficiency (Wang et al., 2011; Danila et al., 2016; Cui, 2021). Kranz structure contains many different forms, but almost all evolved from C3 plants (Sage et al., 2014). Some genera such as Flaveria and Steinchisma contain both C3 and C4 species or some C3-C4 evolutionary intermediates. Recently, many more C3-C4 intermediates have been found (Brown et al., 2005; Marshall et al., 2007; Keerberg et al., 2014; Khoshravesh et al., 2016). It can be seen that some C3-C4 groups led by Flaveria are important resources for studying the evolutionary mechanism of C4 plants, which provides a valuable biological basis for early attempts to C3 to C4 engineering and for studying the evolutionary mechanism of Kranz anatomical structure (Cui, 2021).

## Methods of cultivating high light efficiency plants

### Enhancing the carboxylation of Rubisco

Studies have shown that up to 1/2 of Rubisco’s reactions during photosynthesis are oxygenation reactions (Tcherkez et al., 2006). Therefore, this enzyme is regarded as one of the most important targets to improve the photosynthetic efficiency of C3 plants. Some studies suggest that CCM of cyanobacteria can be introduced into C3 plants (Zarzycki et al., 2013; Hanson et al., 2016). This special CCM can achieve high CO_2_ concentration around Rubisco only through the carboxy matrix in the cell (Lin et al., 2014). Compared to the general mechanism by which C4 plants achieve CO_2_ enrichment through spatial separation of photosynthesis, this CCM mechanism for cyanobacteria is clearly much simpler and thus very promising for C3 crop improvement. Recently, it has been found that by introducing five photosynthetic enzymes from *Zea mays* into specific cells of rice leaves, the minimum C4 cycle can be constructed to achieve the purpose of CO_2_ concentration and have little impact on rice. This also provides another strong evidence for the feasibility of introducing CCM into rice (Ermakova et al., 2021).

### Enhancing the catalytic activity of Rubisco

In addition, the low catalytic activity of Rubisco is also one of the main reasons for the low photosynthetic efficiency of C3 plants. Different from the efficient catalytic ability of other enzymes, Rubisco’s catalytic rate is very slow, and each active site can only catalyze about 3.7 enzymatic reactions per second, while most other enzymes can catalyze more than 100 reactions (Parry et al., 2013). This makes plants need to synthesize Rubisco in large quantities to compensate for their low catalytic capacity. It is estimated that plants need to consume up to 50% of leaf soluble protein and 25% of leaf nitrogen to maintain sufficient photosynthetic rate, which greatly limits the growth of plants (Lin et al., 2014). Therefore, improving the catalytic capacity of Rubisco is also an inevitable problem in cultivating high light efficiency plants.

### Bypassing endogenous photorespiration

Photorespiration has become the most important factor limiting the photosynthetic yield of plants, which can cause 20-50% yield loss of C3 crops (Wang et al., 2020; Nayak et al., 2022). It is one of the effective measures to avoid the loss of fixed carbon by designing and installing the metabolic bypass of photorespiration to inhibit or bypass the endogenous photorespiration. *E. coli* can use glycolate as the sole carbon source and can release CO_2_ while metabolizing (Pellicer et al., 1996). By introducing the glycolic acid metabolism pathway of *E. coli* into crops such as *Arabidopsis* and potato, the researchers made the plants express some or all glycolic acid metabolism related genes, reduce or bypass their own photorespiration, and significantly improve plant photosynthesis (Kebeish et al., 2007; Nolke et al., 2014). The same method was applied to the improvement of cucumber, and it was found that the partial or complete introduction of exogenous glycolic acid pathway increased the biomass by 44.9% and 59%, respectively (Chen et al., 2019).

### Leaf phenotypic improvement

Besides endogenous genetic regulation, leaf phenotype is also an important factor affecting photosynthetic efficiency. More dense leaf veins and smaller and many more stomata contribute to gas exchange and material transport, which is an effective method to improve photosynthetic efficiency (Feldman et al., 2017; Xiong et al., 2022). Furthermore, the size, curl, inclination angle and other structures of leaves all affect the photosynthetic efficiency of plants. Through the comparison between different rice varieties, it was found that the leaves of rice with high photosynthetic efficiency often have the anatomical characteristics of larger mesophyll cells, more chloroplasts, fewer mesophyll cells between adjacent two leaf veins, and larger chloroplast surface area (Mathan et al., 2021). The content of chlorophyll and other photosynthetic pigments is directly related to the photosynthetic potential and primary productivity. It is generally considered that high chlorophyll content is an ideal phenotype. However, some studies have shown that higher chlorophyll may affect the available light of lower leaves and thus affect the photosynthesis of the whole plant. Reducing the antenna size to appropriately reduce the chlorophyll content, on the contrary, contributes to the improvement of PSII efficiency and nitrogen use efficiency (Song et al., 2017; Leister, 2022).

### Prolonging the photosynthetic period of leaves

In rice grains, nearly 80% of the reserve substances come from photosynthesis of leaves after heading (Da Costa et al., 2022). For example, hybrid maize, because of its longer period of photosynthetic activity, still maintains a higher chlorophyll content during senescence relative to normal maize, thereby increasing grain yield (Wu et al., 2016b). This indicated that it was a feasible way to achieve high yield by prolonging the photosynthetic function period of leaves and preventing premature senescence of leaves. Improving the synthesis capacity of chlorophyll, slowing down the senescence and degradation speed of chloroplast, and improving the antioxidant capacity of chloroplast are the main means to ensure the photosynthetic efficiency (Khan et al., 2020; Tang et al., 2020; Yu et al., 2020; Pang et al., 2022). It was found that leaf senescence may be induced by hexose accumulation. By applying different concentrations of sucrose to young and mature leaves, it was found that the electrolyte leakage and malondialdehyde level of leaves increased under high sucrose concentration, resulting in the decrease of leaf photosynthesis (Asim et al., 2022).

## Improve the heat tolerance of plants

In the 21st century, due to the intensification of the greenhouse effect and global warming, extreme weather occurs frequently in the world, of which extreme high temperature has the most serious impact on plants (Zhao et al., 2022). Taking rice as an example, high temperature will not only affect the normal synthesis and processing of protein, change the activities of various enzymes, lead to disorder of energy metabolism and excessive accumulation of reactive oxygen species, but also cause poor development of flower organs, affect normal pollination and fertilization, lead to poor grain development and shortened filling time, thus reduce the quality and nutritional value of grains (Wu et al., 2016a; Prasertthai et al., 2022). A series of negative effects caused serious damage to plant yield (King et al., 2015). It is estimated that the yield of rice will decrease by 3%-8% for every 1°C increase in temperature (Paul et al., 2020). With the continuous change of the global climate, it can be expected that extreme high temperatures will occur more frequently. Therefore, improving the heat tolerance of plants to improve the stable yield is one of the bases to ensure the cultivation of high light efficiency crops.

### Plant response to heat stress (HS)

#### Signal transmission mechanism in plants

Plants need to convert external temperature changes into internal molecular signals to induce their own heat shock response (HSR). In higher plants, photoreceptors including phytochrome and cryptochrome may be the potential temperature sensors of plants (Miyazaki et al., 2015; Jung et al., 2016; Legris et al., 2016; Fujii et al., 2017). These photoreceptors change their activity through temperature changes, participate in the regulation of various temperature signaling pathways or directly regulate the temperature sensitive response of plants Li et al., 2022. In addition, some transcription factors and regulatory elements also have the characteristics that their activity changes with temperature, and also have the potential as temperature sensors. These features include changes in DNA/chromatin structure, variable splicing of mRNA, changes in RNA secondary structure, etc., which have also been reported (Nusinow et al., 2011; Vu et al., 2019; Perrella et al., 2022). However, most of the temperature receptors reported and identified at present are to regulate the morphological change or developmental transition process of plants under warm-temperature environment, and only one case of temperature receptors tolerant to extreme high temperature has been reported (Zhang et al., 2022).

Ca^2+^signaling is considered to be the fastest signal response in plants (Kan and Lin, 2021). HS affects the fluidity of plasma membrane and causes a large amount of internal flow of Ca^2+^ in a short time. It can reach the peak in a short period of 15s, thus rapidly transmitting the external temperature signal (Saidi et al., 2010). The elevated Ca^2+^ level in the cytoplasm induces various stress responses to tolerant high temperature by activating or inhibiting the activities of Ca^2+^/CAM related kinases, phosphatases and transcription factors. In addition, reactive oxygen species (ROS) have also recently been reported to be involved in the heat shock response of plants, which activates the plant’s overall defense mechanism by stimulating stressed tissues to generate a series of responses while transmitting stress signals from stressed to non-stressed areas (Zandalinas et al., 2020).

#### Heat shock protein

When HS occurs, the normal function of the endoplasmic reticulum is affected, resulting in a large accumulation of unfolded and misfolded proteins, resulting in cytotoxicity. Meanwhile, plants will activate the transcription of heat shock factor (HSF) and induce a large number of heat shock proteins (HSPs). This kind of special proteins with molecular chaperone activity can maintain the thermal stability of other proteins and ensure that other proteins perform correct post-translational folding. Some HSPs also play a role in the degradation of abnormal proteins and non-functional proteins (Taipale et al., 2010; Usman et al., 2017; Kang et al., 2022). HSP is considered to be one of the most important defense responses of plants under HS, which can significantly improve the survival rate of plants. Moreover, many HSPs are not only induced by high temperature conditions, but also activated by various other stresses such as hypoxia, drought and heavy metal ions, thus becoming an important part of plant tolerance under various abiotic stresses.

#### HS memory

Due to climate change, high temperature usually occurs temporarily and repeatedly. In order to cope with this situation, plants have the ability to maintain and acquire heat tolerance at the end of HS events, so that plants can respond to repeated HS events more quickly, which is called HS memory (Charng et al., 2006). HS memory includes two types, namely type I and type II (Oberkofler et al., 2021). Both types guarantee that the genes corresponding to them are able to be continuously induced or show an enhancement of re-induction at the end of HS. This physiological response is induced by some key HSFs (such as HSFA2) and involves histone methylation around memory genes and the maintenance of low nucleosome occupancy, thus ensuring its efficient induction (Perrella et al., 2022). HS memory can last up to 5-6 days in *Arabidopsis*, which significantly improves the tolerance of plants to high temperature (Liu et al., 2018).

### Breeding heat tolerant rice by genetic methods

Directional genetic improvement of rice by molecular biology has significant advantages over traditional breeding methods, which can greatly shorten the breeding period and ensure the accurate improvement of target traits. With the help of heat-tolerant germplasm resources including *N22*, various means were used to explore the molecular mechanism of rice HSR and identify relevant genes/QTLs, so as to realize the direct regulation of rice heat response pathway. At present, a large number of heat resistance related genes have been identified, which has provided some help for heat resistance breeding of rice. However, the number of successfully cloned genes is very limited (**Table 1**) (Rabara et al., 2021). Therefore, it is still the focus of rice heat tolerance research to successfully clone the identified heat tolerance genes and make them be used in breeding practice.

**Table 1:** The map-based cloned genes related to heat tolerance in rice.

Gene symbol	MSU	Mapping population	How to clone	Coding product	Regulation	Function	donor	marker	mutation type	Reference
*OgTT1*	LOC_Os03g26970	BC_4_F_2_(6721)	Map-based cloning	α2 subunit of the 26S proteasome	Positive regulation	Eliminate cytotoxic denatured protein	CG14	SSR	Point mutation	(Li et al., 2015)
*TOGR1*	LOC_Os03g46610	F_2_	Map-based cloning	DEAD-box RNA helicase	Positive regulation	Regulate rRNA homeostasis	Zhong Xian 3037	STS, CAPS	Single nucleotide G to T substitution	(Wang et al., 2016a)
*OsCAO1*	LOC_Os10g41780	cross line between *pale green leaf mutant* and *TN1*	Map-based cloning	chlorophyllide an oxygenase 1	Positive regulation	impact leaf senescence	*japonica rice* variety Yunyin	SSR	Point mutations lead to premature termination of translation	(Yang et al., 2016)
*OsBHT*	LOC_Os01g55270	Cheongcheong/Nagdong DH line	Map-based cloning	an Hsps-p23-like calcyclin-binding protein	Positive regulation	Participate in HSR as a molecular chaperone	CNDH75	SSR	Higher expression level	(Park et al., 2020)
*SLG1*	LOC_Os12g39840	F_2_(834 mutants)	Map-based cloning	Cytosolic tRNA 2-thiolation protein 2	Positive regulation	Regulate the level of thiolated tRNA	KY131	whole-genome polymorphic marker	Single-nucleotide substitution resulting in mis-splicing and the formation of multiple transcripts	(Xu et al., 2020)
*HTS1*	LOC_Os04g30760	F_2_(1370 mutants)	Map-based cloning	β-ketoacyl carrier protein reductase	Positive regulation	Maintain membrane stability and chloroplast integrity	Wuyunjing 7	SSR, STS	Single nucleotide substitution	(Chen et al., 2021)
*PSL50*	LOC_Os01g50770	F_2_(170 mutants)	Map-based cloning	A clathrin-associated adaptor protein complex 1 medium subunit μ1 (AP1M1)	Positive regulation	Negatively regulates heat-induced premature leaf senescence	Zhongjian 100	SSR	1-bp deletion mutation	(He et al., 2021)
*HES1*	LOC_Os08g10600	F_2_	Map-based cloning	UDP-N-acetylglucosamine pyrophosphorylase	Positive regulation	play essential roles in maintaining chloroplast function	*indica rice (Oryza sativa)* cv ZF802	SSR	Point mutation led to premature termination	(Xia et al., 2022)
*EMF1*	LOC_Os01g42520	Ethyl methanesulfonate mutagenized population of *Yixiang 1B*	Map-based cloning	A DUF642 protein	Positive regulation	Early flowering	YX1B	SSR	14‐bp deletion	(Xu et al., 2022)
*HTH5*	LOC_Os05g05740	BC_5_F_2_(7648)	Map-based cloning	Pyridoxal phosphate homeostasis protein	Positive regulation	Affect seed setting rate	HHT3	SSR	nucleotide mutation in the promoter region	(Cao et al., 2022)
*OgTT2*	LOC_Os03g29370	BC_5_F_2_(7820)	Map-based cloning	A Gγ subunit and an unknown protein	Negative regulation	Regulate the biosynthesis of wax	HP21	InDel, CAPS	Point mutation led to premature termination	(Kan et al., 2022)
*OgTT3.1*	LOC_Os03g49900	Chromosome segment substitution lines	Map-based cloning	RING-type E3 ligase	Positive regulation	Ubiquitinate *TT3.2* for vacuolar degradation	CG14	InDel, CAPS	one amino acid substitution	(Zhang et al., 2022)
*OgTT3.2*	LOC_Os03g49940	Chromosome segment substitution lines	Map-based cloning	Chloroplast precursor protein	Negative regulation	Accumulation under HS cause chloroplast damage	CG14	InDel, CAPS	one amino acid substitution	(Zhang et al., 2022)

In recent years, with the development of molecular biology, some regulatory elements led by E3 ubiquitin ligase and microRNA have been revealed to participate in the regulation of heat tolerance in rice and other plants (Ning et al., 2011; Song et al., 2013; Cui et al., 2017). Different from the genes/QTLs that directly affect the phenotype of plant traits, these stress response factors cause the decrease of gene expression or the change of protein abundance through regulating various metabolic pathways, and ultimately affect heat tolerance of plants. This undoubtedly provides a new channel for breeding rice with heat tolerance. However, although many heat tolerance related regulatory loci and QTLs have been reported, these studies cannot further improve rice yield (Khan et al., 2019; Rabara et al., 2021). In other words, these studies can only guarantee the stability of rice yield under various high temperature conditions. Due to the rapid growth of the global population, the current grain production has been difficult to meet the future demand. It is difficult to achieve the breeding goal of increasing production simply by improving heat resistance. A few studies have identified some genes/QTLs that simultaneously affect rice heat tolerance and yield, but few of them have been successfully cloned (Wang et al., 2016b; El-Esawi and Alayafi, 2019; Lo et al., 2020). The ultimate goal of heat-resistant breeding is to make the rice yield meet the increasing food demand under future climate conditions. Therefore, it is necessary to take yield improvement into account when studying heat resistance.

### Improve breeding efficiency

Although the current use of marker-assisted selection (MAS) to aggregate the main effect QTL or gene of the target trait has significantly improved the breeding efficiency compared with the traditional methods, heat tolerance of rice is a complex trait controlled by multi minor-effect gene loci. Thus, the aggregation of several major-effect genes alone does not result in a very ideal phenotype, which limits the further improvement of breeding efficiency (Spindel et al., 2015).

Genome selection (GS) refers to a method of selection using high-density molecular genetic markers covering the whole genome to calculate genome estimated breeding value (Meuwissen et al., 2001). This method is still a kind of MAS method in essence, but it has many advantages compared with general MAS breeding (Varshney et al., 2020). First, GS allows early selection without detecting for major effect genes affecting the target trait and frees the reliance of traditional breeding on phenotypic information with increased accuracy. Moreover, GS has successfully realized the identification and selection of many small effect genes, which is more conducive to the improvement of complex traits (Newell and Jannink, 2014). Finally, compared with the traditional MAS method, GS is more effective in improving traits with low heritability and difficult to measure (Yu et al., 2022). It can be seen that if GS can be applied to improve heat tolerance of rice, it will reduce or even get rid of the phenotypic identification, improve the accuracy of selection, further improve the breeding efficiency, and have a broad application prospect.

## Summary

Photosynthesis, as a direct source of plant yield, has received extensive attention. Many studies hope to further enhance the accumulation of plant assimilates by improving photosynthetic efficiency. The photosynthesis of plants is affected by both itself and the environment. Due to the limitation of nitrogen assimilation and source sink, the improvement of plant photosynthetic efficiency by changing the environmental CO_2_ concentration is very limited. It is expected that future research will still focus on the genetic improvement of plants. The improvement of photosynthetic pathway or components and the introduction of C4 pathway are the main research focuses at present. The former has been reported in a large number, but the latter is still at an early stage of exploration. In order to realize plant C3-C4 engineering, the spatial separation of photosynthesis and Kranz structure are the two key problems to be solved, and the utilization of some C3-C4 intermediates point out the promising direction. In addition, with the global warming and the frequent occurrence of extreme weather, high temperature has increasingly become one of the important factors limiting plant yield. HS almost affects all physiological processes of plants, including photosynthesis, and even causes plant death in serious cases. Therefore, cultivating heat tolerant rice under the current climate conditions has become one of the important objectives of breeding. At present, the related genes successfully cloned are limited, which makes it difficult to cultivate heat-resistant rice. Therefore, it will be one of the key problems for future research to apply heat-resistant genes to breeding practice. Moreover, because most of the current studies have little relationship with rice yield traits, different genes/QTLs affecting yield and heat tolerance have to be considered simultaneously in the breeding process, which undoubtedly increases the burden of breeding work and extends the breeding period. It is expected that how to strengthen the relationship between heat tolerance and rice yield will be an important issue to be solved in the future to improve breeding efficiency.

## Author contributions

QS and YX jointly wrote this mini review, XQ helped to revise the manuscript, and JY conceived and guided the writing of the manuscript.

## Funding

This work was financially supported by grants from the National Natural Science Foundation of China (32171931) and the open project from State Key Laboratory of Rice Biology (160102) to JY.

## Conflict of interest

The authors declare that the research was conducted in the absence of any commercial or financial relationships that could be construed as a potential conflict of interest.

## Publisher’s note

All claims expressed in this article are solely those of the authors and do not necessarily represent those of their affiliated organizations, or those of the publisher, the editors and the reviewers. Any product that may be evaluated in this article, or claim that may be made by its manufacturer, is not guaranteed or endorsed by the publisher.
